# Influence of Phenol-Enriched Olive Oils on Human Intestinal Immune Function

**DOI:** 10.3390/nu8040213

**Published:** 2016-04-11

**Authors:** Sandra Martín-Peláez, Olga Castañer, Rosa Solà, María José Motilva, Margarida Castell, Francisco José Pérez-Cano, Montserrat Fitó

**Affiliations:** 1 Cardiovascular Risk and Nutrition Research Group, REGICOR Study Group, Hospital del Mar Research Institute (IMIM), Barcelona 08003, Spain; ocastaner@imim.es (O.C.); mfito@imim.es (M.F.); 2 Spanish Biomedical Research Networking Centre (CIBER), Physiopathology of Obesity and Nutrition (CIBEROBN), Institute of Health Carlos III, Madrid 28029, Spain; 3 Unit of Farmacobiology, Faculty of Medicine and Health Sciences, University Rovira i Virgili, Reus 43201, Spain; rosa.sola@urv.cat; 4 Food Technology Department, UTPV-XaRTA, University of Lleida-Agrotecnio Center, Lleida 25198, Spain; motilva@tecal.udl.cat; 5 Department of Physiology, Faculty of Pharmacy, University of Barcelona, Barcelona 08028, Spain; margaridacastell@ub.edu (M.C.); franciscoperez@ub.edu (F.J.P.-C.); 6 Nutrition and Food Safety Research Institute (INSA-UB), Barcelona 08028, Spain

**Keywords:** olive oil, phenolic compounds, gut microbiota, mucosal immunity, inflammation, IgA-coated bacteria

## Abstract

Olive oil (OO) phenolic compounds (PC) are able to influence gut microbial populations and metabolic output. Our aim was to investigate whether these compounds and changes affect the mucosal immune system. In a randomized, controlled, double blind cross-over human trial, for three weeks, preceded by two-week washout periods, 10 hypercholesterolemic participants ingested 25 mL/day of three raw virgin OO differing in their PC concentration and origin: (1) an OO containing 80 mg PC/kg (VOO); (2) a PC-enriched OO containing 500 mg PC/kg from OO (FVOO); and (3) a PC-enriched OO containing a mixture of 500 mg PC/kg from OO and thyme (1:1, FVOOT). Intestinal immunity (fecal immunoglobulin A (IgA) and IgA-coated bacteria) and inflammation markers (C-reactive protein (CRP) and fecal interleukin 6 (IL-6), tumor necrosis factor α (TNFα) and calprotectin) was analyzed. The ingestion of high amounts of OO PC, as contained in FVOO, tended to increase the proportions of IgA-coated bacteria and increased plasma levels of CRP. However, lower amounts of OO PC (VOO) and the combination of two PC sources (FVOOT) did not show significant effects on the variables investigated. Results indicate a potential stimulation of the immune system with very high doses of OO PC, which should be further investigated.

## 1. Introduction

Adherence to the Mediterranean Diet (MD) has been associated with a reduced risk of cardiovascular mortality, cancer incidence and mortality, as well as a lower incidence of neurodegenerative diseases [1,2,3,4,5]. The MD is characterized by a high intake of olive oil (OO), vegetables, fruit, legumes, and complex carbohydrates with a moderate consumption of fish, and a low-to-moderate amount of red wine during meals [6]. With respect to nutrients, the MD is very rich in phenolic compounds (PC), particularly flavonoids, which are bioactive compounds mainly found in plant foods and plant-derived beverages such as coffee, tea and red wine. In this regard, the PC contained in the MD seem to contribute to the observed beneficial effects due to their antioxidant and anti-inflammatory properties. Of special interest is the influence of PC on the immune system, since it is extremely vulnerable to oxidant and antioxidant balance [7]. The ingestion of PC-rich foods [8,9] or beverages [10] has shown positive effects on the immune system. In this regard, most of the few studies in humans reporting modulatory effects of PC on the immune response have focused on flavonoids [11,12,13,14,15,16,17].

OO, the primary source of fat in the MD, is rich in PC, mainly phenolic acids, but also flavonoids, lignans and others. This phenolic fraction includes bioactive components such as hydroxytyrosol, oleuropein, 3,4-DHPEA-EDA (oleacein), 3,4-DHPEA-EA (oleuropein aglycone), *p*-HPEA-EDA (oleocanthal), luteolin, apigenin (+)-pinoresinol, and (+)-1-acetoxypinoresinol, among others [18]. OO PC contribute to the health effects associated with virgin OO consumption [19,20,21], since they are linked epidemiologically to longevity and also to a lower incidence of chronic diseases, especially with a decrease in the risk of cardiovascular diseases [22].

The mechanisms by which OO PC can support these activities are varied and, most probably, interconnected [23]. It has been recently reported that the ingestion of extra-virgin OO rich in phenolic compounds from OO alone or in combination with other sources of phenolic compounds (*i.e.*, thyme) for three weeks is able to modify gut microbiota populations and their metabolic output [24]. Gut-associated lymphoid tissue (GALT) constitutes the most extensive and complex part of the immune system in the body. The crosstalk between GALT and the microbiota has been shown to be critical for mucosal tissue homeostasis, maintenance of mucosal barrier function and protection against infectious and inflammatory diseases occurring at mucosal sites. The interaction is a complex and tightly regulated process that distinguishes invasive pathogens and innocuous antigens from food and commensal bacteria [25]. During the metabolism of food and xenobiotics such as PC, the host and its gut microbiota co-produce a large number of small molecules, many of which play critical roles in this crosstalk. Some of these signals from commensal intestinal bacteria continuously support the maintenance of mucosal lymphoid tissue [26].

Few sources of PC have been investigated for their influence on the previously mentioned crosstalk [27,28], and their effect on the immune system and level of inflammation. Based on the evidence that OO PC are able to modify the gut bacterial ecosystem [24,29,30], the aim of this study was to investigate whether these PC have an effect on mucosal and systemic immunity. We are not aware of the existence of previous studies in humans on intestinal immunity provided by PC-rich food and, to the best of our knowledge this is the first time that this particular outcome of OO intake in humans has been explored in a randomized, controlled, double blind cross-over trial.

## 2. Materials and Methods

### 2.1. Study Subjects and Design

The present study included a subsample (*n* = 10, five females and five males) from the VOHF (Virgin Olive Oil and HDL (high density lipoprotein) Functionality) study [31]. This was a randomized, controlled, double-blind, crossover clinical trial in which 33 hypercholesterolemic volunteers, aged 35–80 years, ingested a daily dose of 25 mL of three raw virgin OO differing in their PC concentration and origin of PC: (1) a virgin OO naturally containing 80 mg PC/kg (VOO); (2) a PC-enriched virgin OO containing 500 mg PC/kg from OO (FVOO); and (3) a PC-enriched virgin OO containing a mixture of 500 mg PC/kg from OO and thyme, 1:1 (FVOOT). The full phenolic composition of the three oils is presented in Table 1. The rest of the OO components (fat soluble micronutrients -including α-tocopherol, lutein, β-cryptoxanthin, β-carotene- and fatty acids) were the same in type and amounts for the three oils. Intervention periods were of three weeks preceded by two-week washout periods.

Exclusion criteria were the following: BMI > 35 kg/m^2^, smokers, high physical activity (>3000 kcal/day), diabetes, multiple allergies, intestinal diseases, or other diseases or conditions that would worsen adherence to the measurements or treatments. Participants were asked to keep a three-day dietary record at the beginning of the study and after each intervention period, and to maintain their habitual diet, limiting the consumption of PC-rich food throughout the study. To measure biomarkers of adherence, 24 h urine was collected. Blood at fasting state (of at least 10 h) and fecal samples were collected before and after each intervention period. All participants provided written informed consent, and the local institutional ethics committee (Clinical Research Ethical Committee of the Municipal Healthcare Institute, CEIC-IMAS) approved the protocol (CEIC-IMAS 2009/3347/I). The trial was registered with the International Standard Randomized Controlled Trial register (www.controlled- trials.com; ISRCTN77500181).

### 2.2. Fecal Sample Collection and Pre‑Analytical Treatment

Feces were collected and kept under anaerobic conditions as previously described [24] and brought to the laboratory within 2 h after defecation. Feces were diluted with sterile 0.1 M, pH 7.0, phosphate-buffered saline (PBS, Sigma-Aldrich Co. LLC., St. Louis, MO, USA) (1:10, w:w), mixed in a Stomacher 400 (Seward, Thetford, Norfolk, UK) for 2 min and the fecal slurries homogenized. After centrifugation (1300× *g*, 3 min), hexane (Sigma-Aldrich, UK) was added to a proportion of the fecal homogenate supernatant (4:1, *v*/*v*), mixed by inversion for 2 min and removed after centrifugation (15,500× *g*, 5 min) and evaporation. Pellets were washed in filtered sterile PBS and centrifuged (15,500× *g*, 5 min). Afterwards, they were diluted in 1 mL of PBS/glycerol (1:1) and stored at −20 °C until total and IgA coating bacteria analysis.

For the rest of the fecal analytical determinations (immunoglobulin A (IgA), tumor necrosis factor α (TNF-α), interleukin 6 (IL-6), calprotectin), the remaining proportions of the fecal homogenate supernatant were immediately frozen at −80 °C until ELISA analysis.

### 2.3. Dietary Adherence

Urinary hydroxytyrosol sulfate and thymol sulfate were analyzed as compliance markers to the type of OO ingested in 24 h urine by ultra-HPLC–ESI–MS/MS [32]. A three-day dietary record was administered to the participants at baseline and before and after each intervention period. A nutritionist personally advised participants to replace all types of habitually consumed raw fats with the oils provided, and to limit their PC-rich food consumption.

### 2.4. Analysis of C-Reactive Protein (CRP)

Whole blood collected in ethylenediaminetetraacetic acid (EDTA)-coated tubes was used for the analysis of high sensitivity C-reactive protein (hsCRP) by standardized methods in a Cobas Mira Plus autoanalyzer (Roche Diagnostics Systems, Madrid, Spain). Results were expressed as mg/L.

### 2.5. Analysis of Fecal IgA, Cytokines (TNF-α, IL-6) and Calprotectin

The amounts of IgA, IL-6, TNF-α and calprotectin in feces were quantified by ELISA. For IgA, IL-6 and TNF-α, ninety-six-well polystyrene plates (Nunc MaxiSorp, Roskilde, Denmark) were coated and incubated with anti-human IgA (Bethyl Laboratories Inc., Montgomery, TX, USA), IL-6 (BD Biosciences, San Diego, CA, USA) or TNF-α (BD Biosciences) at 3 µg/mL, 2.5 µg/mL and 2 µg/mL, respectively, in carbonate-bicarbonate buffer overnight. After blocking (tris-buffered saline (TBS) containing 1% bovine serum albumin for IgA; PBS containing 10% fetal bovine serum for IL-6 and TNF-α) for 1 h, the plates were washed and appropriate diluted samples and standard dilutions were added (3 h). After washing, horseradish peroxidase conjugated anti-human IgA and biotin-conjugated anti-human IL-6 or TNF-α antibodies were added (10 ng/mL for IgA and 2 µg/mL for IL-6 and TNF-α) and, after 2 h, peroxidase-conjugated ExtrAvidin (4 mg/mL; BD Biosciences) was incubated for 30 min in the case of IL-6 and TNF-α determinations. Finally, the enzyme-substrate reaction was developed and stopped by adding 3 M H_2_SO_4_ (Merck Millipore, Darmstadt, Germany). Absorbances were measured in a microplate photometer (LabSystems Multiskan, Helsinki, Finland) at 492 nm.

Fecal calprotectin was measured by ELISA using a commercially available kit and instructions provided by the manufacturer (Calprotectin, Human, ELISA kit, HK325-02, Hycult Biotech Inc., Uden, The Netherlands).

IgA, IL-6, TNF-α and calprotectin data were interpolated using ASCENT version 2.6 software (Thermo Fisher Scientific, Waltham, MA, USA) into the standard curves, and expressed as ng/mg of the fecal sample for IgA and as ng/g for IL-6, TNF-α and calprotectin results.

### 2.6. Analysis of Fecal Firmicutes/Bacteroidetes Ratio

The ratio Firmicutes/Bacteroidetes (F/B) was calculated from fluorescence *in situ* hybridization (FISH) data from previous studies [24]. The bacterial groups which accounted for Firmicutes were those hybridized by synthetic oligonucleotide probes labelled with the fluorescent Cy3 dye Ato291 [33], Chis150 [34], Erec482 [34], Fprau645 [35], Lab158 [36], Prop853 [37] and Rrec584 [37] probes. The bacterial groups which accounted for Bacteroidetes were those covered by the Bac303 [38] probe.

### 2.7. Determination of Total Fecal Bacteria and IgA-Coating Bacterial Analysis

The proportion of IgA coating bacteria was analyzed as previously described [28]. Briefly, fecal homogenates diluted in PBS/Glycerol were centrifuged (15,500× *g*, 5 min, 4 °C), later PBS/Glycerol was discarded and pellets washed in PBS (15,500× *g*, 5 min, 4 °C). The pellets were resuspended (1/50) in 1% (*v*/*v*) fetal bovine serum (FBS)/PBS. A solution of 50 µL of 1/40 purified mouse anti-human IgA (BD Biosciences) in 1% (*v*/*v*) FBS/PBS was added and incubated for 1 h. After washing with PBS, a solution of 50 µL of 1/100 anti-mouse IgG (whole molecule) R-Phycoerythrin conjugate (Sigma-Aldrich) in 1% (*v*/*v*) FBS/PBS was added and incubated for 1 h in the dark, washed twice with PBS (8000× *g*, 5 min) and resuspended in PBS until analysis. A non-stained mixture of each sample was used as the control. To label total bacteria, the samples were mixed with DAPI (Sigma-Aldrich Co. LLC., St. Louis, MO, USA) before flow cytometry (FCM) analysis as previously described. FCM analysis was performed using a FACSAria SORP sorter (BD Biosciences), based on bacterial morphology according to their forward-scattered light/side-scattered light (FSC/SSC) signal, at the flow cytometry services of the “Centres Científics i Tecnològics” of the University of Barcelona (CCiT-UB). IgA-coating bacteria results were analyzed by using the Summit v4.3 software (Beckman Coulter, Inc., Miami, FL, USA) and expressed as a percentage of bacterial cells labelled with anti-human IgA with respect to the total cell population labelled with DAPI. Total bacteria was expressed as log_10_ bacteria/g dry feces.

### 2.8. Statistical Analysis

The normality of continuous variables was assessed with normal probability plots and the Shapiro-Wilk test. Non-normally distributed variables were log transformed prior to the analysis. Pearson’s correlation analyses were used to evaluate relationships between variables. Paired *t* test was used for intra-intervention comparisons. Adjusted general linear mixed models with a period-by-treatment interaction term were used for inter-intervention comparisons, providing results as adjusted means. *P* ≤ 0.05 was considered significant. Statistical analyses were performed with an R software version 2.11.1.

## 3. Results

### 3.1. General Systemic Parameters and Compliance

Table 2 shows the participants’ general systemic parameters (body weight (BW), body mass index (BMI), waist, glucose, total cholesterol, high density lipoprotein (HDL) cholesterol, low density lipoprotein (LDL) cholesterol, oxidized-LDL, diastolic blood pressure (DBP), systolic blood pressure (SBP)) and urine compliance markers (hydroxytyrosol sulfate and thymol sulfate) before and after each intervention. The daily consumption for three weeks of the intervention oils, neither influenced body weight nor anthropometric measurements (BW, BMI, waist), which remained constant throughout the trial. Glucose levels increased with the consumption of FVOO compared to VOO, although after the three interventions they remained within levels considered normal for healthy people at fasting state (70 to 100 mg/dL). From the lipidic systemic markers analyzed, a decrease in the levels of oxidized LDL after the FVOOT intervention was observed, compared to pre-intervention values (*P* = 0.034). This decrease in ox-LDL after the ingestion of the FVOOT oil was observed in the 100% of the participants. Regarding blood pressure, none of the OO interventions significantly influenced either SBP or DBP values. Results from compliance markers indicated good adherence to the olive oil interventions, which were well tolerated by all participants, and no adverse events were reported.

### 3.2. Effects on Inflammatory Markers

Plasma concentrations of C-reactive protein (CRP), a main marker of systemic inflammation, were influenced by the OO interventions (Table 3). The levels of CRP increased with the consumption of extra virgin OO containing 500 mg/kg of phenolic compounds only from OO origin (FVOO) compared to pre-intervention values (*P* = 0.025), and also compared to extra virgin OO containing 80 mg/kg of PC from OO (VOO, *P* = 0.040) and extra virgin OO containing 500 mg/kg of PC from two origins (FVOOT, *P* = 0.029). The percentages of participants which increased CRP values were 55.6, 77.8, and 62.5 for VOO, FVOO, and FVOOT, respectively.

Fecal calprotectin and the cytokines IL-6 and TNFα were analyzed as intestinal inflammatory markers (Table 3). All the three OO interventions tended to reduce fecal calprotectin concentrations, although differences were not statistically significant. Differences were observed, however, when analyzing variations of calprotectin levels at an individual level. In this regard, the consumption of FVOOT decreased calprotectin levels in 85.7% of the participants, whereas the consumption of FVOO increased them in 66.7% of the participants (*P* = 0.036). From the two cytokines analyzed in feces, we only detected TNFα, the concentrations of which were not influenced by any of the olive oil interventions.

### 3.3. Effects on Intestinal Microbiota

The effects of the intervention oils on the total bacteria and the ratio Firmicutes/Bacteroidetes is shown in Table 4. Olive oil interventions did not affect the amounts of total bacteria in feces. The ratio of Firmicutes/Bacteroidetes for most of the participants increased after VOO (66.7%), whereas it decreased after FVOOT (71.4%). However these differences were not reflected in the mean values, which showed no statistically significant differences.

### 3.4. Effects on Intestinal Immunity

The effects of the intervention oils on the percentage of bacteria coated to IgA and the amount of secreted IgA were also analyzed (Table 4).

**Figure 1:**
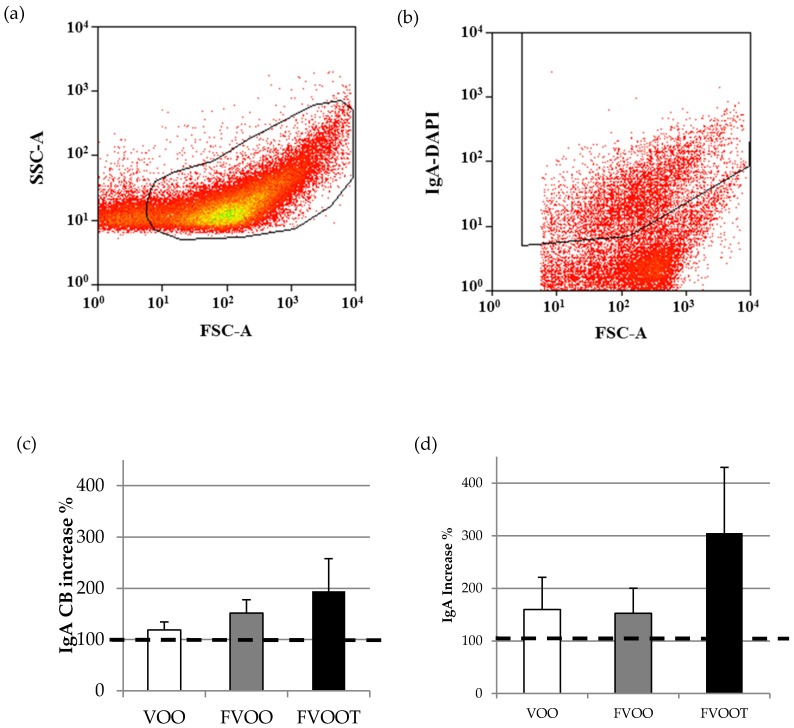
Representative biparametric cytogram showing the (**a**) initial acquisition gate to select the bacterial population according to its forward-scatter characteristic (FSC)/side-scatter characteristic (SSC) signals. (**b**) Cytogram representing the amount of bacteria coated to IgA from the total of bacteria stained with 4′,6-diamidino-2-phenylindole (DAPI). Percentage of increase of fecal IgA coating bacteria (**c**) and IgA (**d**) with each olive oil intervention *versus* pre-intervention values (considering pre-intervention values as 100%). Dietary interventions: 25 mL/day extra virgin olive oil containing: VOO, 80 mg/kg of phenolic compounds (PC) from olive oil; FVOO, 500 mg/kg of PC from olive oil; FVOOT, 250 mg/kg of PC from olive oil and 250 mg/kg from thyme.

Most participants had increased the proportions of IgA-coated bacteria after the three intervention oils. Specifically, 71.4%, 88.9% and 83.3% of the participants for VOO, FVOO and FVOOT, respectively, had higher values of IgA-coated bacteria after the intervention than before. Olive oil interventions tended to increase the proportions of IgA-coated bacteria in feces. The trend was close to significance only after consumption of extra virgin olive oil containing 500 mg/kg of phenolic compounds from one single origin (FVOO, *P* = 0.070). When analyzing increases of IgA-coated bacteria with respect to pre-intervention values (taking pre-values as 100%) (Figure 1), the consumption of VOO increased IgA-coated bacteria by 18.7%, followed by FVOO (51.6%), and FVOOT (94.3%). A similar trend was found for fecal IgA, which increased by 59.7%, 52.8% and 205.3% for VOO, FVOO and FVOOT, respectively.

## 4. Discussion

The present study has investigated the effect of the ingestion of three different olive oils varying in their amount and type of phenolic compounds on the mucosal immune status of hypercholesterolemic subjects. While the ingestion of a virgin olive oil containing 80 mg/kg of olive oil phenolic compounds and the ingestion of a phenol-enriched virgin olive oil containing 500 mg/kg of olive and thyme phenolic compounds (1:1) tended to increase the intestinal IgA response, this did not significantly influence the immunity markers analyzed. In contrast, the ingestion of an OO rich in olive oil phenolic compounds from a unique source (FVOO) showed a higher adhesion of intestinal IgA to gut bacteria, whereas increased a systemic inflammatory marker such as C-reactive protein.

The development and function of the immune system depends on microbial colonization [39]. A regulated cross-talk between commensal bacteria, intestinal epithelial cells and immune cells is also required to maintain intestinal immune homeostasis. IgA is prominently secreted at the intestinal lumen throughout epithelial cells and coats a fraction of the intestinal microbiota. It was estimated that, in healthy conditions, about 36% of intestinal microbiota is covered by IgA, and this proportion can increase up to 69% during inflammatory processes [40], in which it is suggested that IgA identifies inflammatory commensal microbiota that preferentially drive intestinal disease [41]. Our results after FVOO consumption (higher IgA-coating bacteria and CRP) are in line with this relationship between a certain inflammatory status and the increase in microbiota covered by IgA. Although the gut bacteria bound by IgA are poorly characterized, recent studies in murine models suggest exquisite targeting by IgA of distinct commensal bacteria, including segmented filamentous bacteria and *Mucispirillum* [42]. Therefore, changes in commensal intestinal bacteria and/or their metabolic products can stimulate the secretion of IgA to the intestinal lumen. A number of dietary strategies are available for modulating either the composition or metabolic/immunological activity of the human gut microbiota: probiotics, prebiotics and polyphenols, mainly flavonoids, are among the most well established [43]. In this regard, the increase in IgA-coated bacteria generated by the sustained consumption of olive oils studied, but especially that with added phenolic compounds (FVOO), could be due to a change in the composition and metabolic output of gut bacteria, which would stimulate the production and secretion of IgA to the intestinal lumen. A previous study [24] showed no effects in the quantification of the main representative groups of gut bacteria with the consumption of the FVOO. However, qualitative changes in species belonging to the same group could have occurred and stimulated IgA secretion observed in the present work within the framework of the same study. In fact, the microbial metabolic output changed with the consumption of the FVOO, as was indicated by an increase in coprostanone compared to the oil enriched with PC combinations (FVOOT), and by the increased levels of the fecal hydroxytyrosol and dihydroxyphenylacetic acids, compared with pre-intervention values and to VOO, respectively [24]. This hypothetic change in gut bacterial species could be due to a decrease in populations of species more sensitive to high concentrations of olive oil phenolic compounds, which would lead to the expansion of other species that, in turn, would stimulate IgA secretion. On the contrary, the increase in Bifidobacteria reported previously with FVOOT [24], seems to not have a clear effect on IgA secretion because fecal IgA only showed a non-significant trend to increase after such diet. The consumption of other sources of phenolic compounds or flavonoids shows different results regarding IgA and gut bacteria crosstalk. For instance, a cocoa dietary intervention in rats resulted in a decrease in the intestinal IgA secretion and IgA-coating bacteria, with a significant decrease in the proportion of *Bacteroides*, *Clostridium* and *Staphylococcus* genera in the feces of cocoa-fed animals [28].

In human obesity, intestinal microbiota composition has been associated with local and systemic inflammation [44]. In addition, the elevation of IgA-coating bacteria has been associated with a low-grade inflammation in obese people, triggered by the gut microbiota [45]. In our study, the consumption of 25 mL/day of olive oils by participants did not result in an increase of their body weights, and therefore the increase in IgA coating bacteria was not associated with an increase in body weight. The absence of changes in the Firmicutes/Bacteroidetes ratio, which has been associated with obesity or weight loss [46], is in line with these results.

The stimulation of the immune status with high intake of olive oil PC (FVOO) is in consonance with the effects on the systemic inflammatory marker observed, in particular the increased values of plasma CRP. This result apparently disagrees with the antioxidant and anti-inflammatory effects associated with olive oil compounds. In this sense, oleuropein, the most prevalent phenolic component in unriped olives, undergoes hydrolysis during fruit maturation, yielding hydroxytyrosol (2-(3,4-dihydroxyphenyl)ethanol) [47], with recognized antioxidant activity both *in vitro* and *in vivo* [47]. Moreover, it also exerts anti-inflammatory effects by inhibiting lipoxygenase activity and therefore LTB4 production [48], and by inhibiting the production of pro-inflammatory cytokines such as TNFα or IL-1β both by *in vitro* and by animal model approaches. For example, hydroxytyrosol successfully modulated the inflammatory process in models of chronic inflammation as arthritis and colitis mice models, or even in acute models such as the paw edema induced acute inflammation (revised in [22]). In addition, a recent systematic review and meta-analysis shows that the daily consumption of olive oil, ranging approximately between 1 mg and 50 mg, results in a significantly decrease in CRP and IL-6 as compared to controls [49]. However, from the 30 interventions analyzed in the meta-analysis, only two of them reported the amount of PC ingested. In one of them, the amount of PC ingested was lower than the amounts used in our study. Therefore, the unusual high amount of PC included in the olive oil consumed (500 mg/kg) sustained for three weeks, could be responsible for the unexpected effect in CRP after FVOO consumption. It has been stated that high doses of exogenous antioxidant compounds may be toxic, owing to pro-oxidative effects, or their potential to react with beneficial concentrations of reactive oxygen species (ROS) normally present at physiological conditions which are required for optimal cellular functioning [50]. In the other study reported in the meta-analysis, the amount investigated was higher than that we used, but the effects analyzed were at postprandial state [51]. Bogani *et al*. [52] also reported a decrease in CRP after an acute ingestion of high phenol-enriched olive oil (607 mg/kg). In our study, a high amount of PC was ingested for three weeks, which in turn would lead to a high accumulation of antioxidant compounds leading to undesirable effects. Furthermore, if the increase in CRP was mediated by the influence of PC on gut microbiota and GALT crosstalk, postprandial studies with an acute consumption of phenolic compounds would not allow investigating such effects. It has been observed, though, that consumption of high amount of phenolic compounds from different sources could have beneficial effects in this sense. Thus, combined consumption of white wine and extra-virgin olive oil for two weeks decreased the plasma levels of CRP and IL-6 both in patients with chronic kidney disease and healthy individuals [53]. In the same way, the consumption of the same amount of phenolic compounds than FVOO (500 mg/kg) but from different sources (olive oil and thyme, FVOOT) would avoid this undesirable effect. However, we cannot disregard that CRP increases in FVOO groups, which is in line with the increase in intestinal immunity also found, and may also be reflecting the immuno-stimulatory activity of the compounds present in the OO from the gut to the systemic compartment.

## 5. Conclusions

In conclusion, the consumption of a virgin olive oil containing 500 mg/kg of olive oil phenolic compounds increases the proportion of IgA-coated bacteria, which suggests a stimulation of the mucosal immunity at intestinal level. These elevated doses of a unique source of PC also increase the systemic inflammatory marker CRP. Further dose-studies are needed in order to accurately establish the immuno-stimulatory role and the health outcome of these high doses of olive oil phenolic compounds in humans.

## Figures and Tables

**Table 1:** Phenolic composition of the olive oils used in the study.

Phenolic Compounds (mg/25 mL)	Olive Oils ^1^					
	VOO		FVOO		FVOOT	
FLAVONOIDS						
Luteolin	0.04	±0.00	0.18	±0.02	0.21	±0.02
Apigenin	0.02	±0.00	0.06	±0.00	0.10	±0.00
Naringenin	n.d.		n.d.		0.20	±0.02
Eriodictyol	n.d.		n.d.		0.17	±0.01
Thymusin	n.d.		n.d.		1.22	±0.09
Xanthomicrol	n.d.		n.d.		0.53	±0.06
7-methylsudachitin	n.d.		n.d.		0.53	±0.09
*Total flavonoids*	0.06		0.23		2.95	
PHENOLIC ACIDS						
p-hydroxybenzoic acid	n.d.		0.02	±0.00	0.06	±0.00
Vanillic acid	n.d.		0.07	±0.00	0.13	±0.01
Caffeic acid	n.d.		0.00	±0.00	0.06	±0.00
Rosmarinic acid	n.d.		n.d.		0.41	±0.03
*Total phenolic acids*	-		0.09		0.65	
HYDROXYTYROSOL DERIVATES						
Hydroxytyrosol	0.01	±0.00	0.21	±0.02	0.12	±0.00
3,4-DHPEA-AC ^2^	n.d.		0.84	±0.06	0.39	±0.04
3,4-DHPEA-EDA	0.04	±0.00	6.73	±0.37	3.43	±0.29
3,4-DHPEA-EA	0.26	±0.04	0.71	±0.06	0.36	±0.03
*Total hydroxytyrosol derivates*	0.30		8.49		4.30	
LIGNANS						
Pinoresinol	0.05	±0.00	0.12	±0.00	0.10	±0.05
Acetoxipinoresinol	2.47	±0.19	3.66	±0.31	3.24	±0.28
*Total lignans*	2.52		3.78		3.34	
MONOTERPENES						
Thymol	n.d.		n.d.		0.64	±0.05
Carvacrol	n.d.		n.d.		0.23	±0.02
*Total monoterpenes*	-		-		0.86	

Phenolic compounds are expressed as mean ± standard eviation of mg in 25 mL oil/day. ^1^ VOO, 80 mg/kg of OO PC (olive oil phenolic compounds); FVOO, 500 mg/kg of PC from OO; FVOOT, 250 mg/kg of PC from OO and 250 mg/kg from thyme. ^2^ 3,4-DHPEA-AC, 4-(acetoxyethyl)-1,2-dihydroxybenzene; 3,4-DHPEA-EDA, dialdehydic form of elenolic acid linked to hydroxytyrosol; 3,4-DHPEA-EA, oleuropein aglycone. n.d., non-detected.

**Table 2:** Systemic parameters and compliance markers before (B) and after (A) each OO intervention ^1^.

	Olive Oils				*P* ^2^		
		VOO	FVOO	FVOOT	VOO-	VOO-	FVOO-
					FVOO	FVOOT	FVOOT
Body weight	B	80.0 ± 13.77	79.3 ± 14.07	79.4 ± 14.89			
(kg)	A	79.9 ± 14.22	79.5 ± 14.08	80.1 ± 14.37	0.462	0.155	0.420
BMI (kg/m^2^)	B	28.6 ± 4.47	28.3 ± 4.62	28.4 ± 4.89			
	A	28.5 ± 4.66	28.4 ± 4.60	28.6 ± 4.71	0.525	0.209	0.470
Waist (cm)	B	99.6 ± 12.05	99.9 ± 12.60	100.2 ± 10.99			
	A	99.9 ± 12.28	99.9 ± 11.02	99.7 ± 12.38	0.895	0.697	0.795
Glucose	B	95.0 ± 10.59	87.0 ± 8.60	89.8 ± 8.77			
(mg/dL)	A	86.8 ± 7.61	95.3 ± 10.74	90 ± 8.78	0.015	0.211	0.226
Cholesterol	B	210.4 ± 26.01	220 ± 29.42	214.3 ± 37.03			
(mg/dL)	A	207.7 ± 28.81	211.2 ± 23.26	215.2 ± 28.65	0.686	0.811	0.524
HDL (mg/dL)	B	52.0 ± 9.01	51.3 ± 10.29	50.2 ± 10.79			
	A	50.7 ± 9.64	51.7 ± 9.69	51.6 ± 9.94	0.566	0.379	0.750
LDL (mg/dL)	B	133.2 ± 23.66	144.5 ± 22.52	143.3 ± 25.54			
	A	132.9 ± 24.31	134.7 ± 20.18	140.6 ± 20.60	0.448	0.848	0.577
oxidized-LDL	B	43.6 ± 11.35	45.3 ± 6.84	48.7 ± 10.28			
(Units/Liter)	A	43.1 ± 8.70	40.3 ± 6.36	39.8 ± 13.22 *	0.416	0.139	0.483
DBP (mmHg)	B	70.0 ± 8.05	71.9 ± 8.40	71.7 ± 9.19			
	A	72.3 ± 6.38	75.1 ± 11.30	71.6 ± 8.41	0.774	0.455	0.306
SBP (mmHg)	B	126.0 ± 11.19	126.6 ± 10.53	125.5 ± 14.06			
	A	119.7 ± 7.05	122.1 ± 15.90	122.5 ± 12.36	0.707	0.512	0.768
*Compliance markers*							
Hydroxytyrosol	B	25.0 ± 26.44	30.66 ± 25.23	28.17 ± 31.03			
sulfate (µmol/24 h)	A	46.3 ± 28.68	87.66 ± 37.69 *	59.92 ± 47.01 *	0.068	0.578	0.189
Thymol sulfate	B	257.8 ± 317.17	111.9 ± 181.00	289.1 ± 475.26			
(µmol/24 h)	A	96.6 ± 218.91	163.9 ± 206.95	885.0 ± 374.15 *	0.324	0.001	0.016

Values are given as adjusted means ± standard error; *n* = 10 subjects. ^1^ 25 mL/day extra virgin olive oil containing: VOO, 80 mg/kg of phenolic compounds (PC) from olive oil; FVOO, 500 mg/kg of PC from olive oil; FVOOT, 250 mg/kg of PC from olive oil and 250 mg/kg from thyme. ^2^ *P* values for inter-interventions comparisons. * *P* < 0.05 for intra-intervention comparisons. BMI: body mass index; HDL: high density lipoprotein; LDL: low density lipoprotein; DBP: diastolic blood pressure; SBP: systolic blood pressure.

**Table 3:** Inflammatory markers before (B) and after (A) each olive oil intervention ^1^.

Variable		Olive Oils			*P* ^2^		
		VOO	FVOO	FVOOT	VOO-	VOO-	FVOO-
					FVOO	FVOOT	FVOOT
CRP	B	2.0 (0.28, 14.88)	1.4 (0.33, 5.71)	1.9 (0.43, 8.59)			
(mg/L)	A	1.7 (0.33, 8.62)	2.6 (0.51, 13.02) *	1.5 (0.28, 8.01)	0.040	0.866	0.029
IL-6	B	n.d.	n.d.	n.d.			
(ng/g feces)	A	n.d.	n.d.	n.d.	-	-	-
TNF-α	B	28.4 (0.89, 901.86)	28.6 (0.23, 3.4 × 10^3^)	49.6 (0.45, 5.4 × 10^3^)			
(ng/g feces)	A	10.6 (0.51, 216.57)	27.4 (0.04, 2.1 × 10^4^)	41.2 (0.41, 4.1 × 10^3^)	0.558	0.606	0.935
Calprotectin	B	64.3 ± 35.50	82.5 ± 52.42	61.9 ± 41.45			
(ng/g feces)	A	55.8 ± 41.93	59.9 ± 44.67	50.5 ± 35.58	0.603	0.916	0.690

Values are given as adjusted means and confidence intervals, for variables with non-normally distributed data. Values are given as adjusted means ± SE for variables with normally distributed data. *n* = 10. ^1^ 25 mL/day extra virgin OO containing: VOO, 80 mg/kg of phenolic compounds (PC) from OO; FVOO, 500 mg/kg of PC from olive oil; FVOOT, 250 mg/kg of PC from OO and 250 mg/kg from thyme. ^2^ *P* values for inter dietary intervention comparisons. * *P* ≤ 0.05 for intra dietary intervention comparisons. CRP: C-reactive protein; TNF-α: tumor necrosis factor α; IL-6: interleukin 6.

**Table 4:** Markers of intestinal immunity before (B) and after (A) each olive oil intervention ^1^.

Marker		Olive Oils			*P ^2^*		
		VOO ^1^	FVOO	FVOOT	VOO-	VOO-	FVOO-
					FVOO	FVOOT	FVOOT
Total bacteria	B	9.2 ± 0.41	9.1 ± 0.28	9.2 ± 0.18			
(log_10_ bacteria/	A	8.8 ± 0.39 **	9.1 ± 0.36	9.1 ± 0.25	0.030	0.059	0.855
g dry feces)							
Ratio F/B	B	10.6 (1.11, 100.53)	10.22 (0.91, 115.47)	9.7 (0.79, 119.86)			
	A	9.2 (0.86, 99.26)	7.42 (0.72, 75.93)	10.4 (0.97, 111.73)	0.770	0.753	0.538
IgA-CB	B	36.9 ± 15.93	36.46 ± 9.41	38.3 ± 20.86			
(% total bacteria)	A	42.7 ± 19.37	47.62 ± 10.28 *	47.8 ± 16.88	0.565	0.716	0.854
IgA	B	5.8 (0.35, 95.88)	6.2 (0.25, 152.05)	6.2 (0.95, 40.19)			
(ng/mg feces)	A	4.0 (0.21, 78.76)	5.8 (0.29, 116.26)	7.1 (0.77, 64.59)	0.704	0.539	0.799

Values are given as adjusted means and confidence intervals, or as adjusted means of log_10_ bacteria/g dry feces ± SE (for bacterial counts) for variables with non-normally distributed data. Values are given as adjusted means ± SE for variables with normally distributed data. *n* = 10. ^1^ 25 mL/day extra virgin olive oil containing: VOO, 80 mg/kg of phenolic compounds (PC) from olive oil; FVOO, 500mg/kg of PC from olive oil; FVOOT, 250 mg/kg of PC from olive oil and 250 mg/kg from thyme. ^2^ *P* values for inter dietary intervention comparisons. * 0.05 < *P* < 0.1; ** *P* ≤ 0.05 for intra dietary intervention comparisons. IgA: immunoglobulin A; IgA-CB: immunoglobulin A coated bacteria.

## Abbreviations

The following abbreviations are used in this manuscript:

3,4-DHPEA-AC: 4-(acetoxyethyl)-1,2-dihydroxybenzene
3,4-DHPEA-EA: oleuropein aglycone
3,4-DHPEA-EDA: dialdehydic form of elenolic acid linked to hydroxytyrosol
BMI: Body mass index
BW: Body weight
CRP: C-reactive protein
DAPI: 4′,6-diamidino-2-phenylindole
DBP: Diastolic blood pressure
EDTA: Ethylenediaminetetraacetic acid
ELISA: Enzyme-Linked Immunosorbent Assay
F/B: Firmicutes/Bacteroidetes ratio
FCM: Flow cytometry
FISH: Fluorescence *in situ* hybridization
FSC: Forward-scattered light
FVOO: Extra virgin olive oil containing 500 mg/kg of phenolic compounds from olive oil
FVOOT: Extra virgin olive oil containing 250 mg/kg of phenolic compounds from olive oil and 250 mg/kg from thyme
GALT: Gut-associated lymphoid tissue
HDL: High density lipoprotein
HPLC–ESI–MS/MS: High-performance liquid chromatography/electrospray ionization tandem mass spectrometry
IgA: Immunoglobulin A
IL-6: Interleukin 6
LDL: Low density lipoprotein
MD: Mediterranean Diet
OO: Olive oil
ox-LDL: Oxidized low density lipoprotein
PBS: Phosphate Buffered Saline
PC: Phenolic compounds
*p*-HPEA-EDA: Ligstroside-aglycone di-aldehyde
SBP: Systolic blood pressure
SSC: Side-scattered light
TBS: Tris-buffered saline
TNFα: Tumor necrosis factor α
VOHF: Virgin Olive Oil and HDL Functionality
VOO: Extra virgin olive oil containing 80 mg/kg of phenolic compounds from olive oil
